# Suicide Prevention by Peers Offering Recovery Tactics (SUPPORT) for US Veterans With Serious Mental Illness: Community Engagement Approach

**DOI:** 10.2196/56204

**Published:** 2024-05-23

**Authors:** Samantha A Chalker, Jesus Serafez, Yuki Imai, Jeffrey Stinchcomb, Estefany Mendez, Colin A Depp, Elizabeth W Twamley, Karen L Fortuna, Marianne Goodman, Matthew Chinman

**Affiliations:** 1 Veterans Affairs San Diego Healthcare System San Diego, CA United States; 2 Department of Psychiatry University of California, San Diego San Diego, CA United States; 3 Department of Psychiatry Geisel School of Medicine at Dartmouth Hanover, NH United States; 4 James J. Peters Veterans Affairs Medical Center Bronx, NY United States; 5 Icahn School of Medicine at Mount Sinai New York, NY United States; 6 Veterans Affairs Pittsburgh Healthcare System Pittsburgh, PA United States; 7 RAND Corporation Santa Monica, CA United States

**Keywords:** suicide prevention, advisor, veterans, recovery, community, peer specialist, peer support, serious mental illness, participatory design, community engagement, lived experience

## Abstract

**Background:**

Peer specialists are hired, trained, and accredited to share their lived experience of psychiatric illness to support other similar individuals through the recovery process. There are limited data on the role of peer specialists in suicide prevention, including their role in intervention development.

**Objective:**

To better understand peer specialists within the Veterans Health Administration (VHA), we followed partnership community engagement and a formative research approach to intervention development to (1) identify barriers, facilitators, and perceptions of VHA peer specialists delivering a suicide prevention service and (2) develop and refine an intervention curriculum based on an evidence-informed preliminary intervention framework for veterans with serious mental illness (SMI).

**Methods:**

Following the community engagement approach, VHA local and national peer support and mental health leaders, veterans with SMI, and veteran peer specialists met to develop a preliminary intervention framework. Next, VHA peer specialist advisors (n=5) and scientific advisors (n=6) participated in respective advisory boards and met every 2-4 months for more than 18 months via videoconferencing to address study objectives. The process used was a reflexive thematic analysis after each advisory board meeting.

**Results:**

The themes discussed included (1) the desire for suicide prevention training for peer specialists, (2) determining the role of VHA peer specialists in suicide prevention, (3) integration of recovery themes in suicide prevention, and (4) difficulties using safety plans during a crisis. There were no discrepancies in thematic content between advisory boards. Advisor input led to the development of Suicide Prevention by Peers Offering Recovery Tactics (SUPPORT). SUPPORT includes training in general suicide prevention and a peer specialist–delivered intervention for veterans with SMI at an increased suicide risk. This training aims to increase the competence and confidence of peer specialists in suicide prevention and the intervention supports veterans with SMI at an increased suicide risk through their recovery process.

**Conclusions:**

This paper intends to document the procedures taken in suicide prevention intervention development, specifically those led by peer specialists, and to be a source for future research developing and evaluating similar interventions.

**Trial Registration:**

ClinicalTrials.gov NCT05537376; https://classic.clinicaltrials.gov/ct2/show/NCT05537376

## Introduction

Suicide prevention is the top priority for the US Department of Veterans Affairs (VA). The 2023 National Veteran Suicide Prevention Annual Report described increases in veteran suicides between 2020 and 2021 [1]. Moreover, certain groups remain at high risk for suicide. For example, veterans with serious mental illness (SMI, eg, psychosis and bipolar disorders) have more than twice the rate of suicide and death ideation compared with nonveterans with SMI [2] and higher suicide rates compared to the general US and veteran populations [3]. Among veterans who recently used Veterans Health Administration (VHA) services, veterans with bipolar disorder had increased rates of suicide deaths from 2001 to 2021 [1], while those with schizophrenia had increased rates from 2019 to 2020 [4] but an overall decrease in suicide deaths from 2001 to 2021 [1]. All these individuals interacted with the VHA. As such, the VHA may be an ideal space to intervene and prevent future suicides.

There may be a limit to impact and usefulness of current suicide prevention standards of care for those with SMI. In a review of trials with suicide outcomes, researchers found that 61.7% of all trials and 75% of psychotherapy trials *excluded* individuals based on psychosis [5]. Relatedly, those with SMI are difficult to engage in and retain in treatment [6], experience cognitive impairments [7-11], and have limited social supports [12-15]. Therefore, veterans with SMI are an important high-risk group to target for suicide prevention interventions tailored to their unique needs.

An overarching emphasis for psychosocial treatments for SMI in the VHA is “recovery,” a process of change in which individuals strive to build a fulfilling life regardless of challenges stemming from mental health conditions [16]. A vital aspect of the recovery model is the importance of peer support, a nonmanualized form of social support in which people with similar challenges (eg, psychiatric and substance use disorders) help one another by sharing information and perspectives, helping develop problem-solving skills, and serving as successful role models [17].

Peer specialists are individuals who are certified and trained to use their own lived mental health experiences to support others through the recovery process and are paid or unpaid employees of the mental health system [18]. In VHA, peer specialists must be veterans themselves, and the ~1400 currently employed VA-wide are considered a vital part of VHA mental health recovery services [19]. VHA peer specialists are available to work with veterans once they are connected to care and provide recovery-oriented support as an adjunctive service; peer specialists may also provide outreach to veterans not enrolled in VHA. VHA peer specialists’ scope of practice includes modeling recovery and engendering hope, supporting active engagement in treatment, providing step-down recovery support, encouraging skill use, helping veterans advocate for themselves, and connecting veterans to VA and community resources [19]. Recent reviews of peer support services both in and out of VHA have documented a variety of positive outcomes for service users with SMI (eg, reduced inpatient use and improved recovery, hope, empowerment) [18,20], although some studies found little to no impact on outcomes [18,20,21]. Conclusions from these reviews highlight the need for increased methodological rigor in studies including peers.

Peer specialists are a potentially promising but untested adjunct to clinician-delivered suicide prevention. A recent review of peer specialist–based suicide prevention approaches concluded that they are feasible, including no major negative effects [22]. Peer specialists can address hopelessness, shame, burdensomeness, and social isolation, all psychosocial factors associated with suicide risk according to the interpersonal theory of suicide [23]. VHA peer specialists can screen for suicide but are not permitted to conduct comprehensive risk assessments. Furthermore, in VHA, peer specialists are already often working with individuals at high risk for suicide [24]. For example, data from a recent review of all services provided by all VHA peer specialists showed that 8% of the veterans they work with had a “high-risk suicide flag” on their medical record [25]. Qualitative data from peer specialists and clinicians working in a civilian suicide aftercare program indicate that peer specialists positively value working in suicide prevention [26]. Therefore, there is a need to improve the methodological rigor of peer support for SMI as well as systematically develop peer-delivered interventions to decrease suicide risk.

To create a peer-delivered suicide prevention intervention, we applied a formative research approach to intervention development [27]. The primary focus was including veteran peer specialists’ input to allow for equal decision-making with academic researchers in the intervention development [28,29]. We focused on potential role challenges that VHA peer specialists may experience in suicide prevention, including the recovery model of mental health and the intersection with other suicide prevention best practices. In this paper, we describe the results of a series of advisory meetings with the aims to (1) identify barriers, facilitators, and perceptions of VHA peer specialists delivering a suicide prevention service and (2) develop and refine an intervention curriculum based on an evidence-informed preliminary intervention framework.

## Methods

### Study Design

**Figure 1:**
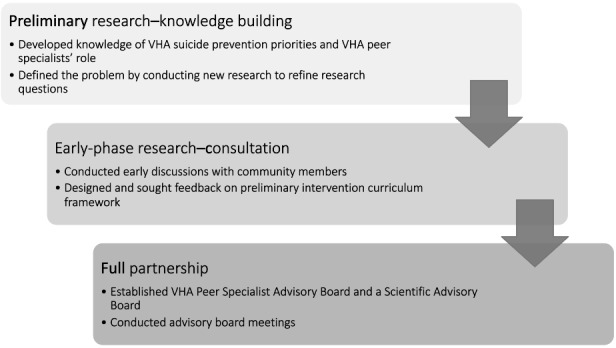
Methodological process of community-engaged partnership intervention development approach. VHA: Veterans Health Administration.

This overall study design is a combination intervention development approach [27] with a specific focus on a partnership through community engagement [29]. Figure 1 displays the methodological process of this study and is in chronological order unless otherwise specified as part of an iterative process.

### Ethical Considerations

The VA San Diego institutional review board (IRB approval H210132) approved the larger research trial in which this paper reports on the initial phase (ClinicalTrials.gov NCT05537376). All advisors provided verbal informed consent. No monetary compensation was provided for participation.

### Preliminary Research—Knowledge Building

#### Developed Knowledge of VHA Suicide Prevention Priorities and VHA Peers Specialists’ Role

In this approach, we first identified gaps in current suicide prevention interventions and standards of care, including understanding the current state of the literature (as documented in the Introduction section), intervention development methodology among those with lived experience [30-32], and community-engaged research to inform intervention development [29,32]. Next, the principal investigator (PI, SAC) held a series of informal discussions with various VHA local and national peer support and mental health leaders, which highlighted encouraging support for VHA peer specialists to be involved in suicide prevention efforts while remaining inside their scope of practice. Simultaneously, the PI sought out and established relationships with scientific experts in suicide prevention, SMI, peer support services, and intervention design. These experts were identified as leaders in their fields by recommendations by others the PI spoke with and some were previously known to the PI. Experts in all areas echoed the same message as VHA leaders. SMI experts specifically emphasized considering cognition when tailoring interventions for individuals with SMI or anyone in an emotional or suicidal crisis. To date, compensatory cognitive strategies [33,34] have not been used to improve learning and recall in the context of suicide prevention interventions but may be crucial as cognitive impairments may limit the ability to recall and engage in preventive behaviors and intervention strategies.

#### Defined the Problem

Preliminary research, based on the new knowledge acquired, was then conducted. This research focused on further understanding the important role of safety planning (ie, a 6-step plan collaboratively completed with a provider and a veteran to identify when the veteran is becoming suicidal and what specifically the veteran can do next [35,36]) as a suicide prevention intervention standard of care given the calls for future research on safety planning [37] and that many trials with suicide-related outcomes exclude individuals on the basis of psychosis [5]. This preliminary research found that increased social support is needed during a suicidal crisis [38,39], that veterans welcome the use of peers in suicide prevention care [40,41], and that improved ability to remember and use one’s safety plan is needed [42-44]. Further conversations were needed to determine whether peers could enhance safety planning.

### Early Phase Research—Consultation

#### Discussions With Community Members

Valuing veteran peer specialists’ perspectives on their potential role in suicide prevention, the PI first met with a group of 5 veteran peer specialists across the country interested in providing input as identified by a community liaison expert. Then, the PI joined the monthly meeting of the 9 local VHA peer specialists via invitation from the local recovery coordinator. Across meetings, a major discussion point was “recovery planning,” the prime example being the Wellness Recovery Action Plan (WRAP) [45]. WRAP is a peer-delivered, evidence-based intervention for those with SMI. WRAP centers on identifying broad warning signs of mental illness, developing wellness or coping tools for functional independence, planning for day-to-day effective living within one’s community, and building a crisis and postcrisis plan. WRAP focuses broadly on mental health and shares aspects with suicide safety planning. Although no formal protocols have been tested to adapt recovery planning for suicidal crises, veteran peer specialists informally shared that they have successfully used WRAP with individuals who are suicidal. Given the added benefit of interventions that directly target suicidal ideation and behaviors [46,47], a suicide-focused, WRAP-inspired plan delivered by peer specialists would allow for a recovery-oriented approach to enhance safety planning for veterans with SMI.

#### Designed and Sought Feedback on Preliminary Intervention Curriculum Framework

Following these conversations, a preliminary intervention curriculum framework was designed. Core concepts included valuing recovery, using wellness tools broadly and when suicidal, setting recovery goals, daily planning development, and learning (ie, cognitive training) strategy identification. This first iteration was reviewed by a VHA peer specialist, and, with verbal consent, the VHA peer specialist then explored the core concepts with 3 veterans with SMI. Both the VHA peer specialist and the veterans with SMI shared that the core concepts were relevant and acceptable to discuss in a peer support appointment. Notably, the VHA peer specialist expressed increased comfort, competence, and confidence in suicide prevention care following review of this intervention framework. Local and national VHA leadership provided feedback on the intervention framework and study design in one-on-one meetings over the study planning period. Leadership feedback remained centered on keeping the service within a VHA peer specialist’s scope of practice.

### Full Partnership

#### Established VHA Peer Specialist Advisory Board and a Scientific Advisory Board

Five of the 9 locally employed peer specialists approached agreed to formally participate on a peer specialist advisory board. These advisors were provided with an informational sheet and provided verbal consent for their participation. All advisors on this board were peer specialists employed by the VHA and provided peer support services in mental health–related clinics and outreach teams. Peer specialist advisors attended eight, 30- to 60-minute meetings via videoconferencing to discuss the 3 themes in aim 1.

Of the scientific experts who provided input in the knowledge development phase, 6 scientific advisors were approached based on the sustained relationship with the PI and the unique area of expertise they championed. They all agreed to participate on the scientific advisory board. The scientific advisors included experts in suicide prevention, SMI, VHA peer support services and implementation, and intervention design and development. The scientific advisors attended one-on-one meetings with the PI and met regularly in small breakout groups every 3-4 months via videoconferencing. Each meeting focused on questions pertaining to the scientific advisors’ expertise area to best use that expertise to enhance the intervention and subsequent implementation.

#### Advisory Board Meetings

The PI moderated all advisory board meetings. Before each meeting, advisory board members reviewed the current intervention material. They could provide written feedback in addition to providing their verbal feedback during the meeting. Discussion questions were posed in the following predetermined key areas in each meeting: (1) scope of practice (eg, how would this intervention change or enhance a peer specialist’s duties?); (2) content (eg, what do you think about a person thinking about suicide setting long-term recovery goals?); (3) intervention design (eg, how many appointments should be provided to cover the material?); (4) suicide prevention interventions (eg, what role should safety planning play in the intervention?); (5) training (eg, how much background suicide prevention information should be provided?); and (6) study design (eg, what role do the peer specialists delivering the intervention play in relation to the research team?). Meetings were audio-recorded with verbal consent. Advisors were not given compensation due to the nature of funding available.

### Reflexive Thematic Analysis Process

Observational notes were collected in real time by 2 research staff members who were in attendance and directly after the meetings by the PI. Audio recordings of the meeting discussions were transcribed. A reflexive thematic analysis process was used after each meeting [48,49]. One research staff member and the PI read a transcript to familiarize themselves with the data. Then initial codes of the first meeting’s transcripts for each advisory board were generated noting these codes using Word’s (Microsoft Corp) comment function. Discrepancies were addressed and then codes were collaboratively determined for the remaining meetings. Codes were added into Excel (Microsoft Corp) and organized by potential theme. Themes were then finalized. Intervention material revisions by the research staff included all feedback and were provided to advisors to review 1 month prior to the next meeting. This process was iterative and discussed with the full research team. The process for each subsequent meeting was repeated.

## Results

### VHA Peer Specialist Advisory Board Contributions

#### Overview

VHA Peer Specialist Advisory Board themes included (1) the desire for suicide prevention training for peer specialists, (2) determining the role of VHA peer specialists in suicide prevention, (3) integration of recovery themes in suicide prevention, and (4) difficulties using a suicide safety plan during a crisis.

#### Desire for Suicide Prevention Training for Peer Specialists

Peer specialist advisors were unclear what they are “allowed” to do when working with an individual at high risk for suicide, specifically when that individual was already known to be at a higher risk; they desired training to address these uncertainties. The peer specialist advisors believed that they did not have the confidence and competence to work with someone who is at a high risk for suicide (eg, “I’m worried I won’t know what to do or say [when someone says they are suicidal].”).

#### Determining the Role of VHA Peer Specialists in Suicide Prevention

The peer specialist advisors were unclear of the role of their direct clinical supervisor when a veteran is already identified to be at an increased risk of suicide (compared with whether the risk was newly identified by the peer specialist, eg, “I’ve been told to just bring my supervisor in at any mention of suicide.”). At the same time, peer specialist advisors indicated that they felt that they could do more for a veteran at an increased risk for suicide instead of immediately bringing in a licensed provider (eg, their clinical supervisor) to address the risk (eg, via a comprehensive suicide risk assessment [48]) if they had the necessary training (eg, “I feel like I can do more for suicidal veterans, but I’m not sure what I am allowed to do.”). Peer specialist advisors believed that they should have more autonomy when it comes to working with veterans at risk for suicide (eg, “We can adapt to what is needed in the moment … that’s what we’re best at.”). These advisors expressed interest in continued participation in future phases of study, including providing informed consent to deliver the intervention and to share their experiences.

#### Integration of Recovery Themes in Suicide Prevention

Unique skills peer specialists bring to a suicidal crisis are discussions of recovery and recovery planning (eg, “…I’ve been there and, even if I don’t have the exact same experience, I can still share my story to show that recovery is possible…”). A suicide-focused recovery plan was welcomed by advisors (paired with the appropriate suicide prevention training) as it (1) is within their scope of practice to complete a recovery plan with a veteran and (2) would provide them an intervention that is focused on suicide when they encounter a veteran at an increased suicide risk. Advisors agreed that this type of plan would be useful for veterans before and after a suicidal crisis, suggesting that this intervention could be for veterans at varying risk levels. Establishing rapport at the beginning of the interaction with veterans, focusing on strengths (eg, “specifically, reasons for living”), and modeling effective communication of suicidal thoughts were desired components to include in this intervention.

Relatedly, in developing the design of this intervention, advisors made a series of requests based on the recovery model. First, they asked for the intervention to follow a similar order as other recovery-oriented interventions they deliver in VHA, such as Whole Health. They stated that the general format should start with psychoeducation, move into inspiring hope, and then focus on recovery goals and social connection through daily action planning. Advisors noted that they liked “the option to have multiple versions” of certain materials. For example, the veteran could choose what format they prefer to use for their learning strategies (eg, post-it notes and mobile phone calendar). Finally, the advisors recommended a “triage approach” of what intervention material to focus on first (ie, asking about suicide) and then a hierarchy of recovery topics to target next in each appointment.

#### Difficulties Using a Suicide Safety Plan During a Crisis

In discussing the current standards of care for suicide prevention, the advisors reported potential difficulties with using suicide safety plans during a crisis based on their own lived experience of using a safety plan. Advisors expressed needing something shorter (eg, “a reminder of just my main reason for living and whom I am going to call”), instead of a 1-page or longer document when in a crisis. Advisors also suggested that this shorter plan should be recovery- and strengths-focused as well as “pocket-sized.” Wallet-sized hard copies and digital phone backgrounds with the pertinent information were discussed to have options to meet the needs of varying veteran preferences. Advisors expressed that while veteran patient treatment manuals are helpful for some, the option of translating any curriculum to memorable subelements may be beneficial especially when in a suicidal crisis. Relatedly, they suggested strategies for reminding a veteran to engage with their plan (eg, “Have you ever thought about the use of cell phones or alarms to help people with their daily goals? It’s something I try to use because I’m really good at getting lost in my mind.”). Ultimately, they requested to not have safety planning play a direct role in the intervention except for reviewing the veteran’s safety plan with them if they indicated that they were at higher suicide risk following VHA mandates.

### Scientific Advisory Board Contributions

The scientific advisors’ recommendations were consistent in many respects to the VHA Peer Specialist Advisory Board’s contributions. They echoed the desire for peer specialists to play a valued role in suicide prevention and supported suicide prevention and intervention training for peer specialists within VHA (ie, theme 1). The scientific advisors focused on the peer specialists’ scope of practice within suicide prevention (ie, theme 2), which shaped the overall study design and outcomes as well as the intervention curriculum. There were no discrepancies between the advisory board’s feedback on the intervention.

In determining the role of peer specialists within suicide prevention (ie, theme 2), scientific advisors discussed the boundaries of VHA peer specialists’ scope of practice to address suicide risk (eg, promoting hope but not providing comprehensive suicide evaluations) and concerns from national advisors regarding the protection of peer specialists in this work. Possible iatrogenic effects to the peer specialists were considered paramount. Scientific advisors suggested peer specialists already hired at VHA as part of the Mental Health Care Line to serve as the peer specialists delivering the intervention in the study. The basis of this suggestion was made on funding availability as well as to further illuminate VHA peer specialists’ roles on site, clinic feasibility, and future broader implementation needs. However, including peer specialists as participants in the study was an ongoing point of debate. Some scientific advisors believed that peer specialists should be treated as any other member of the research team—and therefore not participants in the study. Other advisors as well as the local IRB requested peer specialists delivering the intervention to be considered participants (ie, provide informed consent and data) to better learn about potential iatrogenic effects of peer specialists delivering a suicide prevention intervention. To settle this, both sides of the argument were presented to the VHA Peer Specialist Advisory Board, and it was agreed that peer specialists already hired within VHA will be consented participants as part of the study design and documentation of their roles will be pertinent outcomes to the overall study.

In terms of intervention materials and navigating challenges of current standards of care in suicide prevention (ie, themes 3 and 4), scientific advisors focused primarily on the need for compensatory cognitive strategies to increase salience and recall of intervention materials. Similarly, they provided formatting recommendations for the veteran workbook.

### Preliminary Curriculum

#### Overview

Based on the input from both advisory boards, we developed Suicide Prevention by Peers Offering Recovery Tactics (SUPPORT). The aims of SUPPORT are two-fold: (1) increase competence and confidence of peer specialists in suicide prevention and (2) assist veterans with SMI at increased suicide risk through the recovery process.

#### Training

**Figure 2:**
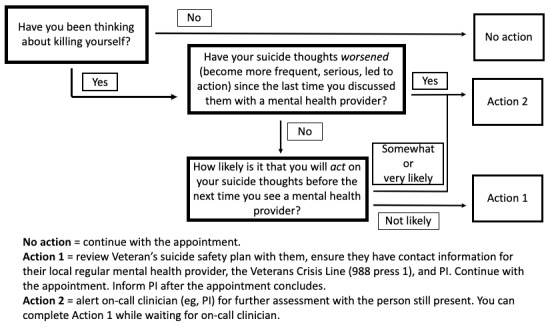
Peer specialist protocol for directly asking about suicidal thoughts and what to do next. PI: principal investigator.

The request for suicide prevention training by advisors led to the development of a training manual tailored to VHA peer specialists including two main sections: (1) a general suicide prevention training that can be a stand-alone training for any peer and (2) a training for VHA peer specialists in how to deliver a recovery-oriented, evidence-informed intervention for veterans with SMI at an increased suicide risk (Multimedia Appendix 1). The complete SUPPORT training includes two 4-hour training days. Adapting from other suicide prevention models for peer specialists [49], Figure 2 demonstrates the procedure in which peer specialists can ask directly about suicidal thoughts and an algorithm for when to incorporate intervention by a licensed provider.

#### Supervision and Consultation

As part of ongoing training, a study consultation group is also provided biweekly (timing based on the peer specialists’ request) after the complete SUPPORT training. The consultation group, comprising the peer specialists delivering SUPPORT, research staff, and a licensed clinical psychologist, serves as a dedicated time to discuss immediate concerns; receive feedback on SUPPORT appointments; discuss and process general concerns, fears, and questions; and discuss implementation or other administrative topics.

#### Intervention Content

**Figure 3:**
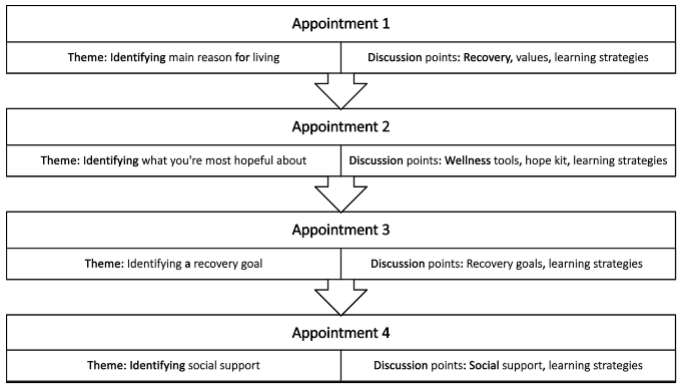
Suicide Prevention by Peers Offering Recovery Tactics intervention preliminary intervention curriculum.

The SUPPORT intervention is designed to promote enhanced personal recovery, quality of life, and connectedness to foster effective management of veterans’ suicidal thoughts and behaviors (Figure 3). As is typical with other peer support services, the SUPPORT intervention augments and complements ongoing care (eg, case management, individual therapy, and psychiatric medication appointments).

After completing a comprehensive mental health evaluation with a licensed VHA provider, the veteran will meet with their peer specialist for approximately four 50-minute appointments to discuss and mutually share elements of value-based living, recovery and action planning, and compensatory cognitive (ie, learning) strategies. Including learning strategies as part of the SUPPORT intervention may improve learning of concepts, memory for intervention elements, prospective memory for symptom self-evaluations, review of intervention material, and promote general functional and social recovery. The final result is pocket-sized hard copy or digital set of 4 reminders for living (ie, the veteran’s main reason for living, what the veteran is most hopeful about, the veteran’s recovery goal, and who the veteran is going to call in a suicidal crisis).

## Discussion

### Principal Findings

Over 18 months, the research team built trust and relationships with peer specialists and scientific advisors across the United States to design a suicide prevention intervention for veterans with SMI using the recovery model. Partnership engagement consisted of contacting potential partners via email to determine interest, building relationships with potential partners, creating 2 separate advisory boards, and meeting separately with both advisory boards to include their input and equal decision-making in the intervention development process. A peer specialist–delivered suicide prevention intervention and a suicide prevention training for VHA peer specialists were developed. This work adds to the literature of lived experience–driven intervention design and development in suicide prevention [30,31]. The steps reported here are intended to document the procedures taken in suicide prevention intervention development, specifically those led by peer specialists, and to be a source for future research developing and evaluating similar interventions.

### Lessons Learned

This research paper depicts lessons learned, but 3 additional lessons are worthy of discussion. The first focuses on staffing. Peer specialists, clinical supervisors, and leadership changes are necessary considerations in VHA training and intervention development work. This study saw turnover of local leadership, leading to delays in the community engagement process due to the uncertain structure of local peer support services. This process will continue to be carefully documented to support related work throughout the remainder of the study. Second, the research funding for this project precludes compensation of VHA employees for study activities. That is, peer specialists employed by VHA—the target population of advisors and deliverers of the intervention—were not eligible for study compensation. Other than monetary compensation, such as time off, should be considered. Third, limited models for advisor engagement exist [50], especially in suicide prevention intervention development [30,51]. Although there are relevant models to draw from in other areas [28,32,52], some of which focus on important diverse and underserved populations [53], and there is a need for clear documentation of partnership engagement in this intersection.

### Limitations

While this study adds to the community engagement literature in suicide prevention intervention development studies, it has limitations. The advisory boards included a small number of individuals who were self-selected and, therefore, may differ from the larger population the study aims to serve. Due to IRB constraints, the advisory board excluded veterans who were not peer specialists. To rectify this, veterans recruited as participants in the implementation phase will participate in qualitative interviews to include their perspectives in refining the intervention. Moreover, while initial advisors included veteran peer specialists outside of VHA, the final advisory board is made up of only VHA-employed peer specialists. Therefore, these findings may not generalize to other community or clinical settings where peer specialists are less common or may have different roles and levels of interaction with patients.

### Conclusions

As Watling et al [30] suggested, a combined methodology is presented here. To further refine these materials, a 2-phase study design that continues to involve these advisory boards is underway. In the remaining portion of phase 1, the research team will train VHA peer specialists via an open pilot trial and continue to monitor the role of the peer specialists as research team members and study participants. Individual semistructured qualitative interviews of the peer specialist and veteran participants will be conducted, and materials will be revised based on these interviews and ongoing input from both advisory boards. The open pilot trial of phase 1 is actively recruiting as of August 2023. This partnership approach to intervention development champions the crucial elements of including voices with lived experience of suicidal thoughts and behaviors in research design, testing, and implementation.

## Appendix

Multimedia Appendix 1 SUPPORT training manual preliminary curriculum for veteran certified peer specialists.

## Abbreviations

IRB: institutional review board
PI: principal investigator
SMI: serious mental illness
SUPPORT: Suicide Prevention by Peers Offering Recovery Tactics
VA: Veterans Affairs
VHA: Veterans Health Administration
WRAP: Wellness Recovery Action Plan

## References

[1] (2023). 2023 National Veteran Suicide Prevention annual report. Office of Mental Health and Suicide Prevention.

[2] Jahn DR, Muralidharan A, Drapalski AL, Brown CH, Fang LJ, Lucksted A (2018). Differences in suicide and death ideation among veterans and nonveterans with serious mental illness. Psychol Serv.

[3] Aslan M, Radhakrishnan K, Rajeevan N, Sueiro M, Goulet JL, Li Y, Depp C, Concato J, Harvey PD (2020). Suicidal ideation, behavior, and mortality in male and female US Veterans with severe mental illness. J Affect Disord.

[4] (2022). 2022 National Veteran Suicide Prevention annual report. Office of Mental Health and Suicide Prevention.

[5] Villa J, Ehret BC, Depp CA (2020). Systematic review of the inclusion of people with psychosis in suicide-specific clinical trials. Crisis.

[6] Dixon LB, Holoshitz Y, Nossel I (2016). Treatment engagement of individuals experiencing mental illness: review and update. World Psychiatry.

[7] Stergiopoulos V, Cusi A, Bekele T, Skosireva A, Latimer E, Schütz C, Fernando I, Rourke SB (2015). Neurocognitive impairment in a large sample of homeless adults with mental illness. Acta Psychiatr Scand.

[8] Velligan DI, Mahurin RK, Diamond PL, Hazleton BC, Eckert SL, Miller AL (1997). The functional significance of symptomatology and cognitive function in schizophrenia. Schizophr Res.

[9] Twamley EW, Doshi RR, Nayak GV, Palmer BW, Golshan S, Heaton RK, Patterson TL, Jeste DV (2002). Generalized cognitive impairments, ability to perform everyday tasks, and level of independence in community living situations of older patients with psychosis. Am J Psychiatry.

[10] Green MF, Kern RS, Heaton RK (2004). Longitudinal studies of cognition and functional outcome in schizophrenia: implications for MATRICS. Schizophr Res.

[11] Green MF (1996). What are the functional consequences of neurocognitive deficits in schizophrenia?. Am J Psychiatry.

[12] Kilbourne AM, McCarthy JF, Post EP, Welsh D, Blow FC (2007). Social support among veterans with serious mental illness. Soc Psychiatry Psychiatr Epidemiol.

[13] Chronister J, Chou CC, Kwan KLK, Lawton M, Silver K (2015). The meaning of social support for persons with serious mental illness. Rehabil Psychol.

[14] Hendryx M, Green CA, Perrin NA (2009). Social support, activities, and recovery from serious mental illness: STARS study findings. J Behav Health Serv Res.

[15] Corrigan PW, Phelan SM (2004). Social support and recovery in people with serious mental illnesses. Community Ment Health J.

[16] (2012). SAMHSA's working definition of recovery. Substance Abuse and Mental Health Services Administration.

[17] Solomon P (2004). Peer support/peer provided services underlying processes, benefits, and critical ingredients. Psychiatr Rehabil J.

[18] Chinman M, George P, Dougherty RH, Daniels AS, Ghose SS, Swift A, Delphin-Rittmon ME (2014). Peer support services for individuals with serious mental illnesses: assessing the evidence. Psychiatr Serv.

[19] (2019). VHA directive 1163: psychosocial rehabilitation and recovery services. Department of Veterans Affairs, Veterans Health Administration.

[20] Lloyd-Evans B, Mayo-Wilson E, Harrison B, Istead H, Brown E, Pilling S, Johnson S, Kendall T (2014). A systematic review and meta-analysis of randomised controlled trials of peer support for people with severe mental illness. BMC Psychiatry.

[21] Chien WT, Clifton AV, Zhao S, Lui S (2019). Peer support for people with schizophrenia or other serious mental illness. Cochrane Database Syst Rev.

[22] Bowersox NW, Jagusch J, Garlick J, Chen JI, Pfeiffer PN (2021). Peer-based interventions targeting suicide prevention: a scoping review. Am J Community Psychol.

[23] Joiner TE, Van Orden KA, Witte TK, Selby EA, Ribeiro JD, Lewis R, Rudd MD (2009). Main predictions of the interpersonal-psychological theory of suicidal behavior: empirical tests in two samples of young adults. J Abnorm Psychol.

[24] Cook JA, Copeland ME, Jonikas JA, Hamilton MM, Razzano LA, Grey DD, Floyd CB, Hudson WB, Macfarlane RT, Carter TM, Boyd S (2012). Results of a randomized controlled trial of mental illness self-management using wellness recovery action planning. Schizophr Bull.

[25] Bowersox NW (2019). Peer providers in the Veterans Health Administration: summary of providers, care, and veterans served, FY2015-FY2018Q2. Department of Veterans Affairs.

[26] Van Zanden B, Bliokas V (2022). Taking the next step: a qualitative study examining processes of change in a suicide prevention program incorporating peer-workers. Psychol Serv.

[27] O'Cathain A, Croot L, Sworn K, Duncan E, Rousseau N, Turner K, Yardley L, Hoddinott P (2019). Taxonomy of approaches to developing interventions to improve health: a systematic methods overview. Pilot Feasibility Stud.

[28] Fleurence R, Selby JV, Odom-Walker K, Hunt G, Meltzer D, Slutsky JR, Yancy C (2013). How the Patient-Centered Outcomes Research Institute is engaging patients and others in shaping its research agenda. Health Aff (Millwood).

[29] Principles of community engagement (2nd ed), NIH publication no. 11-7782. Agency for Toxic Substances and Disease Registry.

[30] Watling D, Preece M, Hawgood J, Bloomfield S, Kõlves K (2022). Developing an intervention for suicide prevention: a rapid review of lived experience involvement. Arch Suicide Res.

[31] Schlichthorst M, Ozols I, Reifels L, Morgan A (2020). Lived experience peer support programs for suicide prevention: a systematic scoping review. Int J Ment Health Syst.

[32] Fortuna K, Barr P, Goldstein C, Walker R, Brewer L, Zagaria A, Bartels S (2019). Application of community-engaged research to inform the development and implementation of a peer-delivered mobile health intervention for adults with serious mental illness. J Particip Med.

[33] Twamley EW, Vella L, Burton CZ, Heaton RK, Jeste DV (2012). Compensatory cognitive training for psychosis: effects in a randomized controlled trial. J Clin Psychiatry.

[34] Twamley EW, Thomas KR, Burton CZ, Vella L, Jeste DV, Heaton RK, McGurk SR (2019). Compensatory cognitive training for people with severe mental illnesses in supported employment: a randomized controlled trial. Schizophr Res.

[35] Stanley B, Brown GK, Karlin BE, Kemp JE, Vonbergen HA (2008). Safety plan treatment manual to reduce suicide risk: veteran version. United States Department of Veterans Affairs.

[36] Stanley B, Brown GK, Brenner LA, Galfalvy HC, Currier GW, Knox KL, Chaudhury SR, Bush AL, Green KL (2018). Comparison of the safety planning intervention with follow-up vs usual care of suicidal patients treated in the emergency department. JAMA Psychiatry.

[37] (2019). VA/DoD clinical practice guideline for the assessment and management of patients at risk for suicide. Department of Veterans Affairs and Department of Defense.

[38] Chalker SA, Parrish EM, Ceren CSM, Depp CA, Goodman M, Doran N (2023). Predictive importance of social contacts on U.S. veteran suicide safety plans. Psychiatr Serv.

[39] Chalker SA, Parrish EM, Ceren CSM, Depp CA, Ilgen MA, Goodman M, Twamley EW, Doran N (2022). Crisis service utilization following completion of a suicide safety plan for veterans with and without affective and nonaffective psychosis. J Psychiatr Res.

[40] Chalker SA (202). U.S. Veteran interest in peer specialists' help to enhance suicide safety plans: understanding Veteran perspectives on safety plan engagement. Association of Cognitive and Behavioral Therapies.

[41] Wilson MP, Waliski A, Thompson RG (2022). Feasibility of peer-delivered suicide safety planning in the emergency department: results from a pilot trial. Psychiatr Serv.

[42] Parrish EM, Quynh A, Scott V, Chalker SA, Chang C, Kamarsu S, Twamley EW, Depp CA (2023). Suicide safety plan self-knowledge in serious mental illness: psychiatric symptom correlates and effects of brief intervention. Community Ment Health J.

[43] Kayman DJ, Goldstein MF, Dixon L, Goodman M (2015). Perspectives of suicidal veterans on safety planning: findings from a pilot study. Crisis.

[44] Matthieu MM, Morissette SB, Clafferty S, Degutis L, Oliver CM, Adkins DA, DeBeer BB (2023). Veteran experiences with suicide ideation, suicide attempt, and social support in safety planning within the Department of Veterans Affairs. Mil Med.

[45] Canacott L, Moghaddam N, Tickle A (2019). Is the Wellness Recovery Action Plan (WRAP) efficacious for improving personal and clinical recovery outcomes? A systematic review and meta-analysis. Psychiatr Rehabil J.

[46] Calati R, Courtet P (2016). Is psychotherapy effective for reducing suicide attempt and non-suicidal self-injury rates? Meta-analysis and meta-regression of literature data. J Psychiatr Res.

[47] Meerwijk EL, Parekh A, Oquendo MA, Allen IE, Franck LS, Lee KA (2016). Direct versus indirect psychosocial and behavioural interventions to prevent suicide and suicide attempts: a systematic review and meta-analysis. Lancet Psychiatry.

[48] King D, Lavigne JE (2012). OPERATION S.A.V.E.: suicide prevention training for front-line employees in the U.S. Department of Veterans Health Affairs. Frontiers in Suicide Risk.

[49] Pfeiffer PN, King C, Ilgen M, Ganoczy D, Clive R, Garlick J, Abraham K, Kim HM, Vega E, Ahmedani B, Valenstein M (2019). Development and pilot study of a suicide prevention intervention delivered by peer support specialists. Psychol Serv.

[50] Kujala J, Sachs S, Leinonen H, Heikkinen A, Laude D (2022). Stakeholder engagement: past, present, and future. Bus Soc.

[51] Bornheimer LA, Li Verdugo J, Holzworth J, Im V, Smith FN, Sliwa H, Taylor SF, King CA, Florence T, Tarrier N, Himle JA (2022). Modifying a cognitive behavioral suicide prevention treatment for adults with schizophrenia spectrum disorders in community mental health. Psychiatry Res.

[52] Shelef DQ, Rand C, Streisand R, Horn IB, Yadav K, Stewart L, Fousheé N, Waters D, Teach SJ (2016). Using stakeholder engagement to develop a patient-centered pediatric asthma intervention. J Allergy Clin Immunol.

[53] Vastine A, Gittelsohn J, Ethelbah B, Anliker J, Caballero B (2005). Formative research and stakeholder participation in intervention development. Am J Health Behav.

