# Supplementary material for: Suicide Prevention by Peers Offering Recovery Tactics (SUPPORT) for US Veterans With Serious Mental Illness: Community Engagement Approach
Source: J Particip Med. 2024 May 23;16:e56204. doi: 10.2196/56204 (PMC11157181; doi:10.2196/56204)
Supplement: Multimedia Appendix 1 [file jopm_v16i1e56204_app1.docx]

| **SUPPORT 101** | **SUPPORT Plan Training** | **Appendix** |
| --- | --- | --- |
| **Foundational Knowledge**: prevalence, risk factors, warning signs, theories of developing suicidal thoughts and the transition to acting on those thoughts | **Completed SUPPORT Plan Examples:** Hard copy and digital phone background examples are provided to highlight unique presentations of the material to encourage personalization of the intervention material | **Important Contact Information:** Space to list contact information for emergencies, support, and administration questions |
| **Peer Specialists in Suicide Prevention:** scope of practice defined within suicide prevention | **Consultation Group:** Information on the weekly consultation group for Peer Specialists that are providing SUPPORT, which includes time to receive feedback from fidelity ratings, discuss worries and implementation issues, and discuss their Veterans | **Documentation:** Specific instructions on how to document suicide risk screening, a VHA-specific note template, and information on reducing malpractice liability |
| **Suicide Safety & Risk Management Protocol Topics:** suicide risk/safety screening within scope of practice, crisis intervention/de-escalation techniques, collaborative care and referral process | **Delivery Guidance:** Peer Specialists practice challenging scenarios and then partner with another attendee to real play each section in the SUPPORT Veteran Workbook | **Resources:** National and local resources specific to the Peer Specialist |
| **Self-Care Tools Topics:** managing worries related to working with someone at risk for suicide, setting boundaries, suicide postvention | **Appointment Guides:** unique to each appointment and provide comprehensive information for what to do before, during, and after each appointment | **References:** All cited references listed |
| **Communication Tools Topics:** appropriate terms, communication style, active listening, decreasing stigma, ethical considerations, cultural considerations |  |  |
